# Direct Observation of Long-Chain Branches in a Low-Density Polyethylene

**DOI:** 10.1038/s41598-019-46035-9

**Published:** 2019-07-05

**Authors:** Ken-ichi Shinohara, Masahiro Yanagisawa, Yuu Makida

**Affiliations:** 1 School of Materials Science, Japan Advanced Institute of Science and Technology (JAIST), Nomi Ishikawa, 923-1292 Japan; 2 Advanced Materials Development Laboratory, Sumitomo Chemical Co., Ltd., 2-1 Kitasode, Sodegaura Chiba, 299-0295 Japan

**Keywords:** Polymer characterization, Imaging techniques

## Abstract

Low-density polyethylene (LDPE) has short-chain branch (SCB) and long-chain branch (LCB). In particular, the influence of the structure of LCBs on polymer properties is remarkable; however, it has been difficult to precisely analyze LCB structures. In this study, we measured the chain length of LCBs and the distance between branch points of LDPE by atomic force microscopy. Consequently, three LCBs were confirmed in a main chain of 162 nm, and their length were measured as 10, 31, and 18 nm. The positions of the LCBs were 33, 70, and 78 nm from the main-chain end.

## Introduction

The properties of a polymer change significantly depending on the structure of the polymer chain, particularly, with branched structures, depending on the number of branches and the length of the branch^1–5^. However, the long-chain branch (LCB) structure of polyethylene was unclear, due particularly to the complex polymer structure and the limitations of its analysis methods. Thus, in this study, we aimed to directly observe the LCB structures in low-density polyethylene (LDPE). Specifically, single-molecule imaging was performed by an atomic force microscopy, which improved the structural observation of the polymer chain^6–11^ and allowed direct observation of the number and length of LCBs in LDPE, the positions of the branch points, and the distance between them.

## Experimental

### LDPE sample

LDPE F200-0 produced by Sumitomo Chemical Co., Ltd. (Tokyo, Japan) was used as a sample material. This LDPE was prepared by a tubular process. The LDPE sample was fractionated according to its molecular weight using a tailor-made fractionation system produced by Tosoh Co. (Tokyo, Japan). The system comprises of a manual injector, an oven, a column filled with glass beads, valves and two pumps equipped with vacuum degassers. The oven temperature was set to 125 °C. Xylene (good solvent) and 2-ethoxyethanol (poor solvent) were used as the components of the mobile phase. The xylene concentration in the mobile phase gradually increased, and the sample for atomic force microscope (AFM) observation was collected at a xylene concentration of 59.1–59.6 vol%. The average molecular weights (*M*_w_ and *M*_n_) and molecular weight distribution (MWD) were estimated with an HLC-8121GPC/HT GPC (gel permeation chromatography) or SEC (size exclusion chromatography) system (Tosoh) with three GMH6-HT columns (Tosoh). The SEC system was operated at 140 °C with a flow rate of 1.0 mL/min. The solvent and the eluent used for the analysis were *o*-dichlorobenzene (ODCB) stabilized with 500 ppm of 2,6-di-*tert*-butyl-*p*-cresol (BHT). The concentration of the sample solution and the injection volume were 1.0 mg/mL and 0.3 mL, respectively. Calibration of the system was performed with narrow MWD polystyrene (PS) standards obtained from Tosoh. To transform the molecular weight values of PS to those of PE, the Q-factor^12^ of 41.3 (PS) and 17.7 (PE) was used. The absolute weight-average molecular weight (*M*_w_) and branching index *g*′ (*M*) (the ratio of the intrinsic viscosities of branched and linear polymer at the same molecular weight) were estimated using a GPCIR SEC (GPC) system (Polymer Char, Spain) with three PLgel Olexis columns (Agilent Technologies, CA, USA), equipped with a HELEOS 8 multi-angle laser light scattering detector (MALLS, Wyatt Technology, CA, USA), a viscometer (Visc, Polymer Char) and an IR5 infrared detector (Polymer Char). Here the SEC-MALLS system provided the structural information of a polymer chain in dilute solution^13,14^. This SEC system was operated at 160 °C with a flow rate of 1 mL/min. The solvent and the eluent used for the analysis was 1,2,4-trichlorobenzene (TCB) stabilized with 300 ppm of BHT. The concentration of the sample solution and the injection volume were 2 mg/mL and 0.4 mL, respectively. NIST 1475a (a linear PE standard material) was used as a linear reference for calculating *g*′(*M*). The effect of short-chain branches (SCBs) on *g*′(*M*) was compensated by the method of Sholte *et al*.^15^. The number of branching points in the molecule *n* was obtained using the following equation^16^:

$$g^{\prime} {(M)}^{1/b}={[{(1+n/7)}^{1/2}+4n/9\pi ]}^{-1/2}$$

Here, *b* is a constant relating to the structure of branched polymers. As the most suitable *b* value from our SEC-MALLS-Visc experiments on LDPE samples, we used *b* = 0.9 for the calculation. The weight-average number (*n*_w_) of LCBs in single polymer molecule was also estimated.

The ^13^C-NMR analytical results for LCBs (equal to or longer than C_6_), as *n* (average number of LCBs in a single molecule) or the LCB/1000 C values, and SCBs (shorter than C_6_) as the SCB/1000 C values were estimated using an AVANCE 600 NMR spectrometer (Bruker Co., MA, USA). The measurement conditions and calculation methods are available in the literature^17,18^.

### AFM imaging

A freshly cleaved mica surface of the muscovite substrate (Nilaco, Tokyo, Japan) was obtained using adhesive tape, and any adsorbed water on the mica surface was removed by rinsing with dehydrated THF in dry air (RH < 25%). The AFM sample was prepared by spin-casting (1,500 rpm) hot xylene dilute solution (120 °C, 1 μL) of LDPE onto a mica substrate in dry air. If the dilute polymer solution is only cast/dried on the substrate, the polymer chains easily aggregate to form globules, so the above technique is essential. We altered the specifications of a fast-scanning AFM (NVB500, Olympus, Tokyo, Japan) in dynamic (tapping) mode to observe isolated polymer chains using a cantilever (BL-AC10EGS, BL-AC10DS, Olympus, Japan or USC-F1.2-k0.15, NanoWorld AG, Switzerland)^7,8^. Single-molecule imaging of a polymer was performed by AFM at 25 ± 1 °C in an organic solvent. We verified that decamethyltetrasiloxane (DMTS; TCI) was useful as an observation solvent for AFM imaging of single chains. As the interaction between the polymer chain and mica substrate also depends on its affinity to observation solvent, a solvent suitable for observing the isolated polymer chain on mica was chosen.

To investigate the affinity of solvents, we used the Hansen solubility parameter (HSP)^19^. The Hansen solubility parameters (*δ*) were calculated as follows.

$${\delta }^{2}={\delta }_{d}^{2}+{\delta }_{p}^{2}+{\delta }_{h}^{2}$$

Here, *δ*_*d*_, *δ*_*p*_, and *δ*_*h*_ are the dispersion, electrostatic, and hydrogen-bond components of *δ*, respectively.

Polyethylene (PE instead of LDPE): *δ*_*d*_ = 16.2, *δ*_*p*_ = 2.1, *δ*_*h*_ = 2.4

DMTS: *δ*_*d*_ = 11.7, *δ*_*p*_ = 2.4, *δ*_*h*_ = 0

The HSP distance (*Ra*) between PE and DMTS was also calculated.

$$\begin{array}{rcl}{(Ra)}^{2} & = & 4{[{\delta }_{d({\rm{PE}})}-{\delta }_{d({\rm{DMTS}})}]}^{2}+{[{\delta }_{p({\rm{PE}})}-{\delta }_{p({\rm{DMTS}})}]}^{2}+{[{\delta }_{h({\rm{PE}})}-{\delta }_{h({\rm{DMTS}})}]}^{2}\\ Ra & = & 9.32\end{array}$$

### All-atom molecular dynamics simulation

All-atom molecular dynamics (MD) simulations were performed with the Forcite module of the BIOVIA Materials Studio 2018 (Dassault Systèmes BIOVIA, San Diego, CA, USA) on a supercomputer system (PRIMERGY CX2570 M4, Fujitsu, Tokyo, Japan). See the Supplementary Information for more details.

## Results and Discussion

**Figure 1:**
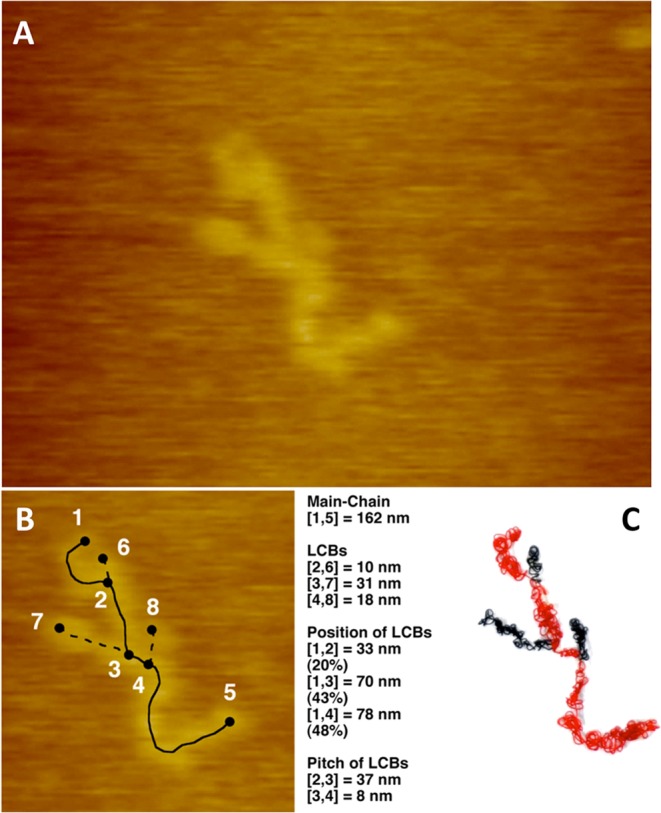
Direct measurement of LCB in a tubular LDPE (F200-0 fractionated). **(A)** AFM image of a single molecule of LDPE on mica in DMTS at 25 °C. X: 279 nm, Y: 209 nm, Z: 18 nm. **(B)** Length of each chain of LDPE. **(C)** A wire model of self-shrinking structure of polymer chain of LDPE. Main chain: red wire. LCB: black wire. The model was created to be one tenth of the length of the extended chain based on AFM observation (**B**), MD simulation (Fig. S1), and the molecular weight determined by SEC-MALLS-Visc experiments (see Fig. 2).

Single-molecule imaging and structural analysis results for LDPE (fractionated sample, *M*_w_: 1.99 × 10^5^, *M*_w_/*M*_n_: 1.2) produced by the tubular method are shown. The structure of single LDPE molecule was directly observed in DMTS on a mica substrate by AFM (Fig. 1A). The length of a LCB in a single molecule, the position of the branch point, and the branching point interval were directly observed (Fig. 1B).

**Figure 2:**
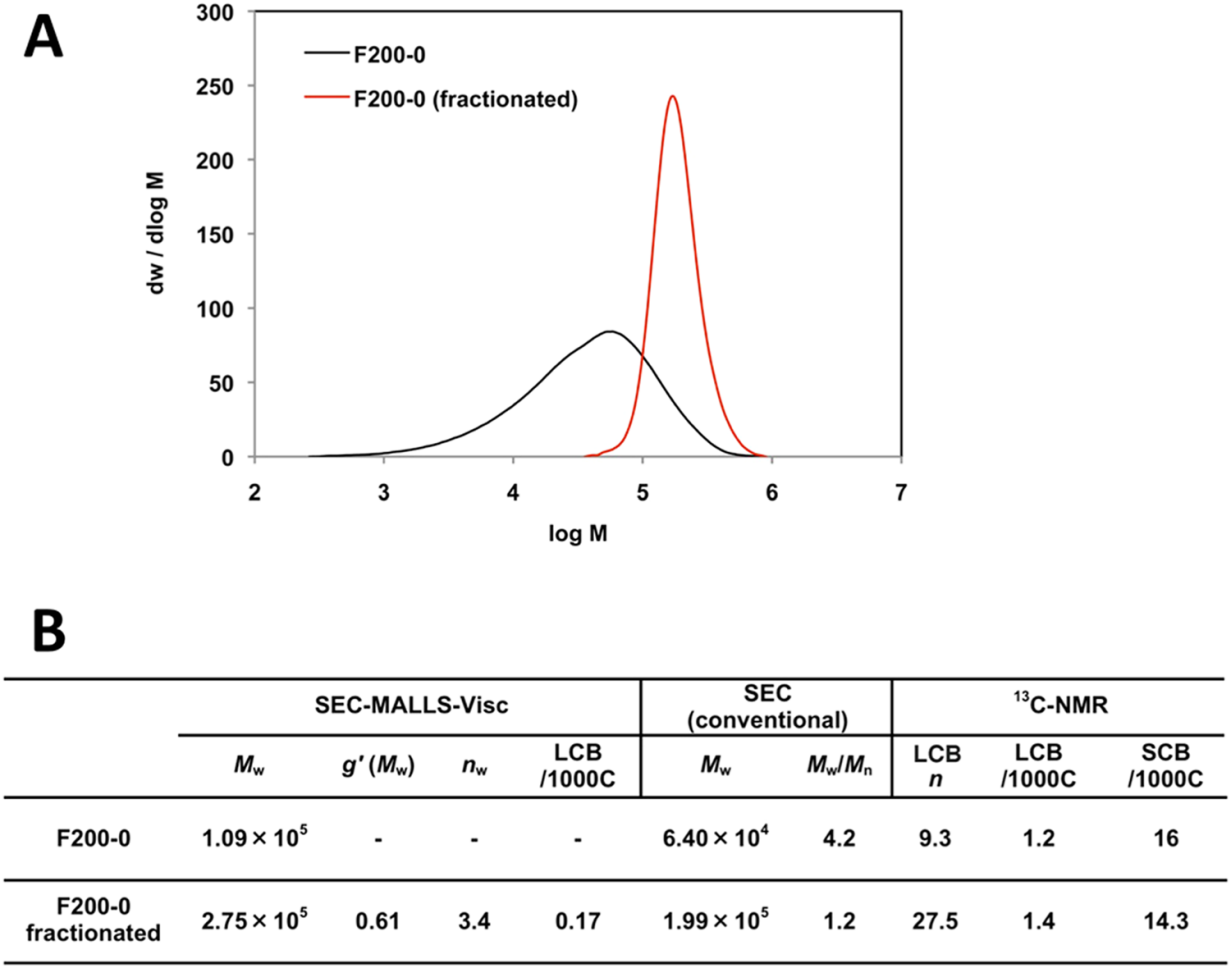
**(A)** SEC chart of LDPE (F200-0) and the fractionated sample, which was used for AFM imaging. **(B)** Analytical results for SEC-MALLS-Visc, SEC (conventional), and ^13^C-NMR (see the experimental section for details).

**Figure 3:**
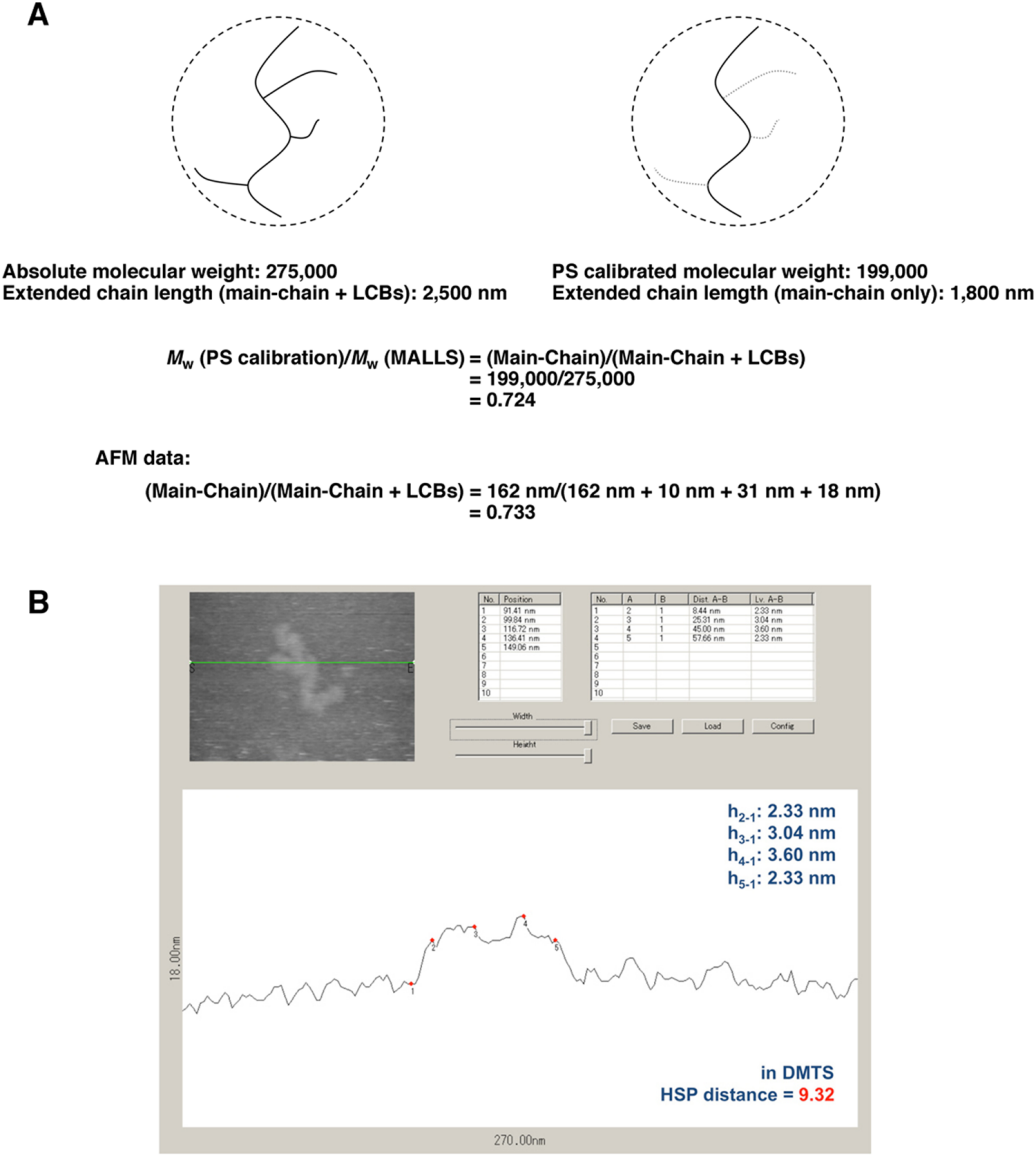
The validity of the value measured by AFM imaging. **(A)** Comparison of SEC and AFM data based on molecular weight and the chain length. The PS-calibrated molecular weight and the extended chain length of the sample were calculated for linear PE by the Q-factor method^12^. (**B**) Line profile of single LDPE molecule on mica in DMTS at 25 °C. The structure with this molecular chain height of 2–3 nm was believed to be self-shrinking (see Fig. 1C).

According to the conventional SEC-MALLS-Visc analytical method, the presence of an average of 3.4 LCBs in a single molecule of this polymer is estimated (*n*_w_ in Fig. 2B). Single-molecule imaging by AFM allowed successful observation of LCB, and the molecular size confirmed that this was a single molecule of this LDPE. Moreover, three LCBs in the main chain of 162 nm were confirmed, measuring 10, 31, and 18 nm in length. The LCBs were located 33, 70, and 78 nm from the chain end, that is, the positions of the branching points. The structure with a molecular chain height of 2–3 nm (Fig. 3B) was believed to be self-shrinking (Fig. 1C). A molecular model of 200-mer LDPE having a LCB that formed a self-shrinking structure in DMTS was verified by the all-atom MD simulation (Fig. S1 and Movie S1).

The validity of this method was investigated by comparing the value obtained by AFM imaging with that determined by the conventional SEC-MALLS-Visc method. This sample polymer is a fraction component of tubular LDPE (Fig. 2A, see experimental section for details). As shown in Fig. 2B, *M*_w_ (PS calibration) indicates a PS-calibrated molecular weight (molecular weight converted to PE), corresponding to the molecular weight of the main chain only (PS: polystyrene, PE: polyethylene). This is because the radius of gyration (*R*_g_) of a single polymer chain in solution is roughly determined by the main chain, and the molecular weight reflecting the main chain is measured (Fig. 3A). In the fractional component of the AFM sample, the value calculated by dividing *M*_w_ (PS calibration) by the absolute molecular weight *M*_w_ (MALLS) obtained by adding the molecular weights of the main-chain and the branch chain is, *M*_w_ (PS calibration)/*M*_w_ (MALLS) = (Main Chain)/(Main Chain + LCBs) = 199,000/275,000 = 0.724 (value 1). However, the length measured by AFM (Fig. 1B) is calculated as (Main Chain)/(Main Chain + LCBs) = 162 nm/(162 nm + 10 nm + 31 nm + 18 nm) = 0.733 (value 2). Value 1 and 2 were similar, confirming the validity of the value obtained by AFM imaging. The extended chain length of the main chain was estimated to be 1,800 nm using SEC, while it was 162 nm according to the AFM measurement. Thus, we propose a self-shrinking model in which the wire is one tenth of the length (Fig. 1C). Similar self-shrinking phenomena of a LDPE chain in DMTS have been confirmed even in all-atom MD simulations (Fig. S1, Movie S1).

Based on the line profile, the molecular height was 2.8 ± 0.6 nm (Fig. 3B). However, ^13^C NMR measurement indicates that approximately one SCB is introduced per 100 carbons (Fig. 2B); thus, so the crystal structure formation is limited, and the folded structure is considered to be partial in the isolated chain at the solid-liquid interface. The structure having this molecular chain height of 2–3 nm was believed to be a self-shrinking and formed from a series of small globules (Fig. 1B).

Moreover, the radius of gyration (*R*_g_) of a single LDPE molecule in a good solvent is 36 nm as measured by MALLS analysis, and the spread from the center of gravity of the molecular chain in the average structure lies within a sphere of a diameter 72 nm, which is close to the size measured by AFM (Fig. 1A).

The above results indicate that the structure measured by AFM is based on the polymer chain skeleton, and that the globule is formed locally. As the solvent for observation is DMTS (HSP distance with PE is 9.32), which is a relatively low solubility to polyethylene, it was believed that small globules were formed.

A wire model (Fig. 1C) that rationally describes all measurements including the chain length (Fig. 1B), line profile (Fig. 3B), and chromatographic results (Fig. 2B) was created. In conclusion, it was demonstrated that LCBs can be measured directly.

This method can be applied both to measuring LCBs in LDPE and elucidating of various branched polymer structures.

## Supplementary information

Supplementary information

Supplementary Movie S1
