# Supplementary material for: Direct Observation of Long-Chain Branches in a Low-Density Polyethylene
Source: Sci Rep. 2019 Jul 5;9:9791. doi: 10.1038/s41598-019-46035-9 (PMC6611765; doi:10.1038/s41598-019-46035-9)
Supplement: Supplementary file 1 — Supplementary information [file 41598_2019_46035_MOESM1_ESM.docx]

**Supplementary Information for**

**Direct Observation of Long-Chain Branches in a Low-Density Polyethylene**

Ken-ichi Shinohara,^1^* Masahiro Yanagisawa,^2^ Yuu Makida^1^

^1^ School of Materials Science, Japan Advanced Institute of Science and Technology (JAIST), Nomi, Ishikawa 923-1292, Japan

^2^ Advanced Materials Development Laboratory, Sumitomo Chemical Co., Ltd., 2-1 Kitasode, Sodegaura, Chiba 299-0295, Japan

* Corresponding author: shinoken@jaist.ac.jp

**Contents:**

**1) AFM imaging**

**2) All-atom molecular dynamics simulation**

**3) Supplementary data of AFM imaging**

**Captions:**

**Chart 1.** AFM head with a fluid cell

**Fig. S1.** Snapshots of all-atom MD simulation of a 200-mer LDPE (F200-0) model having a LCB in DMTS at 600 K. (A) 0 ns, (B) 2.5 ns

**Fig. S2.** Direct measurement of LCB in a tubular LDPE. **(A)** AFM image of a single molecule of LDPE on mica in DMTS at 25°C. X: 300 nm, Y: 225 nm, Z: 18 nm. **(B)** Length of each chain of LDPE.

**Fig. S3.** Direct measurement of LCB in a tubular LDPE. **(A)** AFM image of a single molecule of LDPE on mica in *n*-octylbenzene at 25°C. X: 290 nm, Y: 218 nm, Z: 7.2 nm. **(B)** Length of each chain of LDPE.

**Movie S1.** Movie of all-atom MD simulation of a 200-mer LDPE (F200-0) model having a LCB in DMTS at 600 K

**1) AFM imaging.** A freshly cleaved mica surface of the muscovite substrate (Nilaco, Tokyo, Japan) was obtained using adhesive tape, and any adsorbed water on the mica surface was removed by rinsing with dehydrated THF in dry air (RH < 25%). The atomic force microscope (AFM) sample was prepared by spin-casting (1,500 rpm) hot xylene dilute solution (120°C, 1 μL) of LDPE onto a mica substrate in dry air. If the dilute polymer solution is only cast/dried on the substrate, the polymer chains easily aggregate to form globules, so the above technique is essential.

**Chart S1.** AFM head with a fluid cell


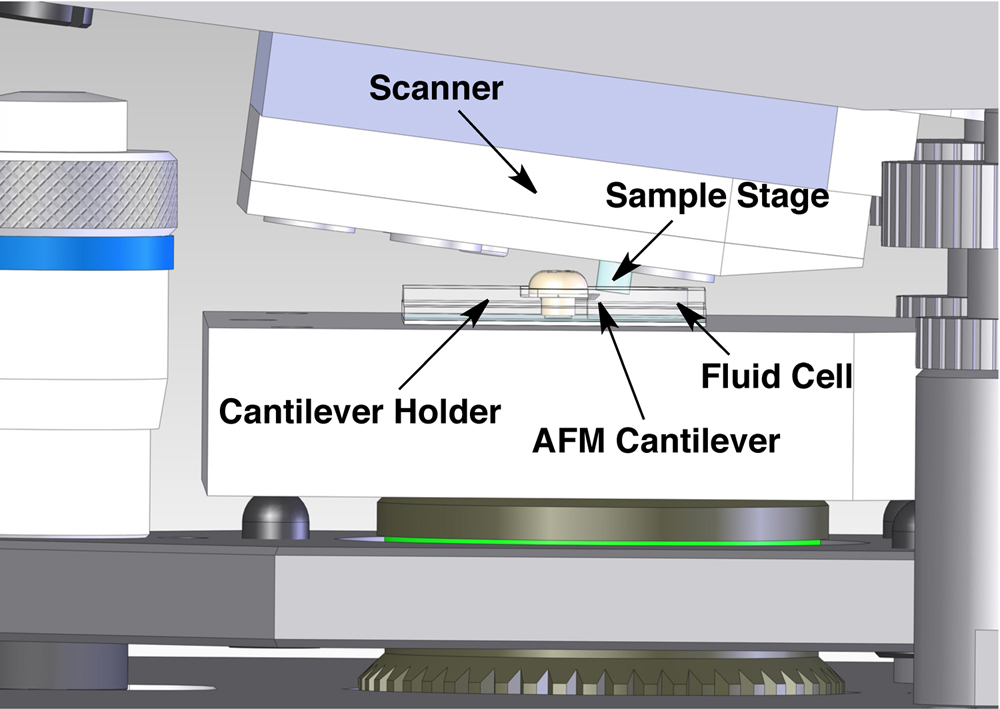


We modified the specifications of a fast-scanning AFM (NVB500, Olympus, Tokyo, Japan) in dynamic (tapping) mode to observe isolated polymer chains. The ultra small cantilever having a low spring constant of around 0.1 N/m and high resonance frequency of over 1 MHz in air was used (BL-AC10EGS, BL-AC10DS, Olympus, Japan or USC-F1.2-k0.15, NanoWorld AG, Switzerland).^1,2)^ A fast-scanning AFM offers outstanding performance for observing the structure of single-molecules in aqueous solution for biological study.^3,4)^ However, we modified the AFM in order to use even in the organic solvent, allowing successful imaging of the single molecular structure of a polymer chain. Chart S1 shows the AFM head with a fluid cell (designed by using the CAD software: SOLIDWORKS, Dassault Systèmes SolidWorks Corporation, Waltham, MA, USA) for imaging in an organic solvent.

Single-molecule imaging of a polymer was performed by AFM at 25 ± 1°C in an organic solvent. In addition to decamethyltetrasiloxane (DMTS; TCI) (Fig. 1, Fig. S2), we confirmed that *n*-octylbenzene (Tokyo Chemical Industry (TCI), Tokyo, Japan) (Fig. S3) is also useful as an observation solvent for single-chain AFM imaging. Because the interaction between the polymer chain and a mica substrate also depends on the affinity to observation solvent, a solvent suitable for observing the isolated polymer chain on mica was selected.

**2) All-atom molecular dynamics simulation.** All-atom molecular dynamics (MD) simulations were carried out using the Forcite module of the BIOVIA Materials Studio 2018 (Dassault Systèmes BIOVIA, San Diego, CA, USA) on a supercomputer system (PRIMERGY CX2570 M4, Fujitsu, Tokyo, Japan). A 200-mer model of LDPE F200-0 having three LCBs was build by use of the Polymer Builder module. The short-chain branch (SCB) content was set to that of the F200-0. The MD cell was built by means of usual procedure of the Amorphous Cell module. The MD cell length and angle were (a = 200 å, b = 80 Å, c = 80 Å) and (α = 90º, β = 90º, γ = 90º), respectively. Here, the 200-mer model was put in the center of the cell, and the solvent molecules of DMTS were packed in the cell at density of 0.854 g cm^−3^. Sequentially, the geometry of the MD cell was optimized. Simulation in the NVT ensemble (constant number of atoms, volume and temperature) was conducted at 298 K for 20 ps (time step of 0.2-fs, 100,000 steps) and the NPT ensemble (constant number of atoms, pressure and temperature) was conducted at pressure of 1.013 × 10^−4^ GPa and at 298 K for 80 ps (time step of 1.0-fs, 80,000 steps) to equilibrate the MD cell. The Nose thermostat was used to control the temperature. The Berendsen barostat was used to control the pressure. After the equilibration at 298 K, simulation in the NVT ensemble (constant number of atoms, volume and temperature) was conducted at 600 K for 20 ps (time step of 1.0-fs, 20,000 steps) as the thermal equilibration. After the equilibration, simulation in the NVT ensemble (constant number of atoms, volume and temperature) was conducted at 600 K for 2.5 ns (time step of 1.0-fs, 2,500,000 steps) as the production run. The COMPASS II (ver. 1.2) forcefield was used, and the charges were assigned by the forcefield. The MD snapshots were indicated in Fig. S1 and Movie S1.


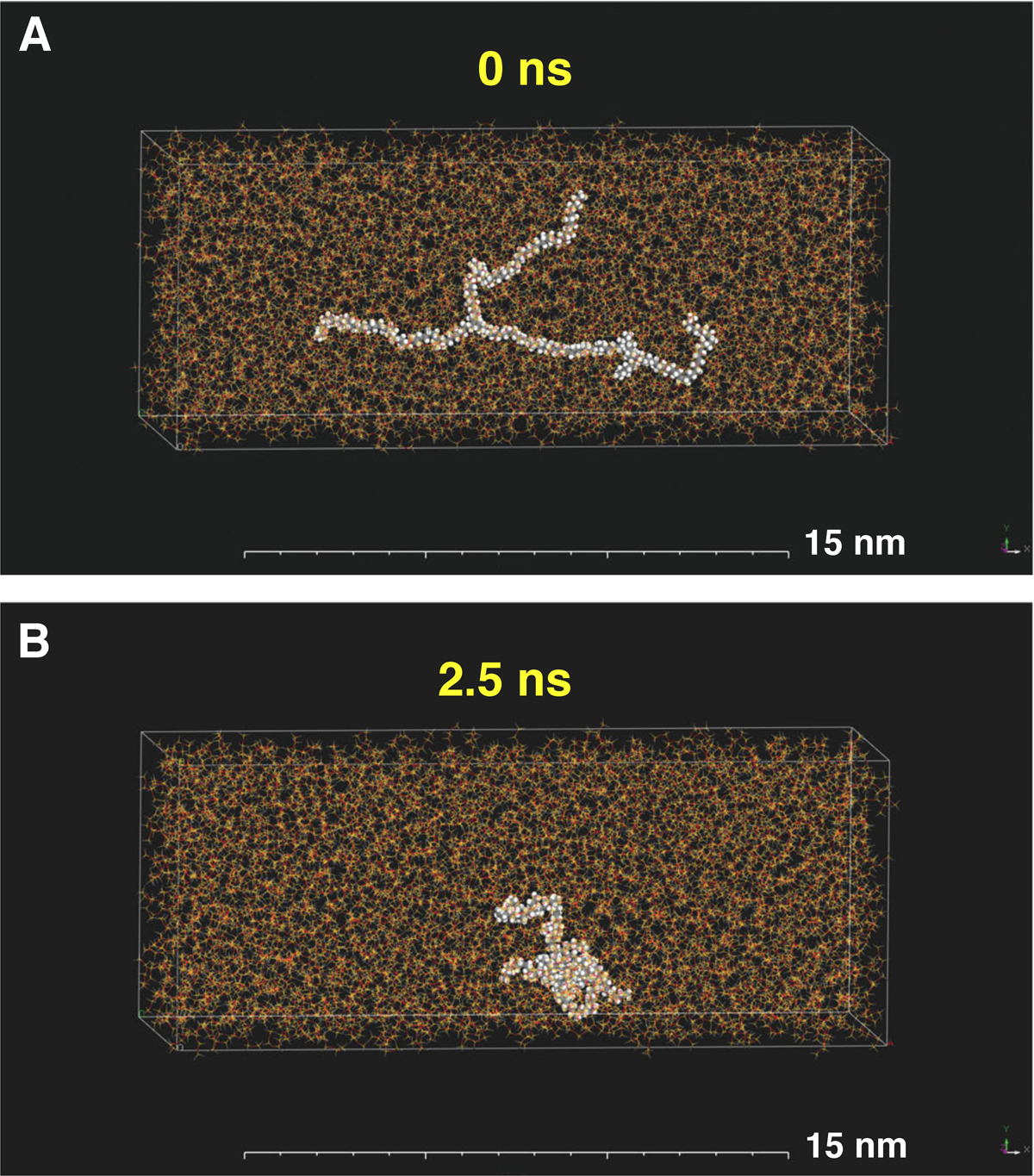


**Fig. S1.** Snapshots of all-atom MD simulation of a 200-mer LDPE (F200-0) model having a LCB in DMTS at 600 K. (A) 0 ns, (B) 2.5 ns. (Movie S1)

The self-shrinking process was confirmed to be about one-tenth of the chain length and small globule of ca. 2 nm size.

**3) Supplementary data of AFM imaging:** Direct observation and measurement results of LCBs in a tubular LDPE are shown in Fig. S2 and S3.

The structure of single polymer chain of LDPE was directly observed in DMTS on mica substrate by AFM (Fig. S2A). The length measured by AFM (Fig. S2B) is calculated as (Main Chain)/(Main Chain + LCBs) = 161 nm/(161 nm + 18 nm + 32 nm) = 0.763.

**
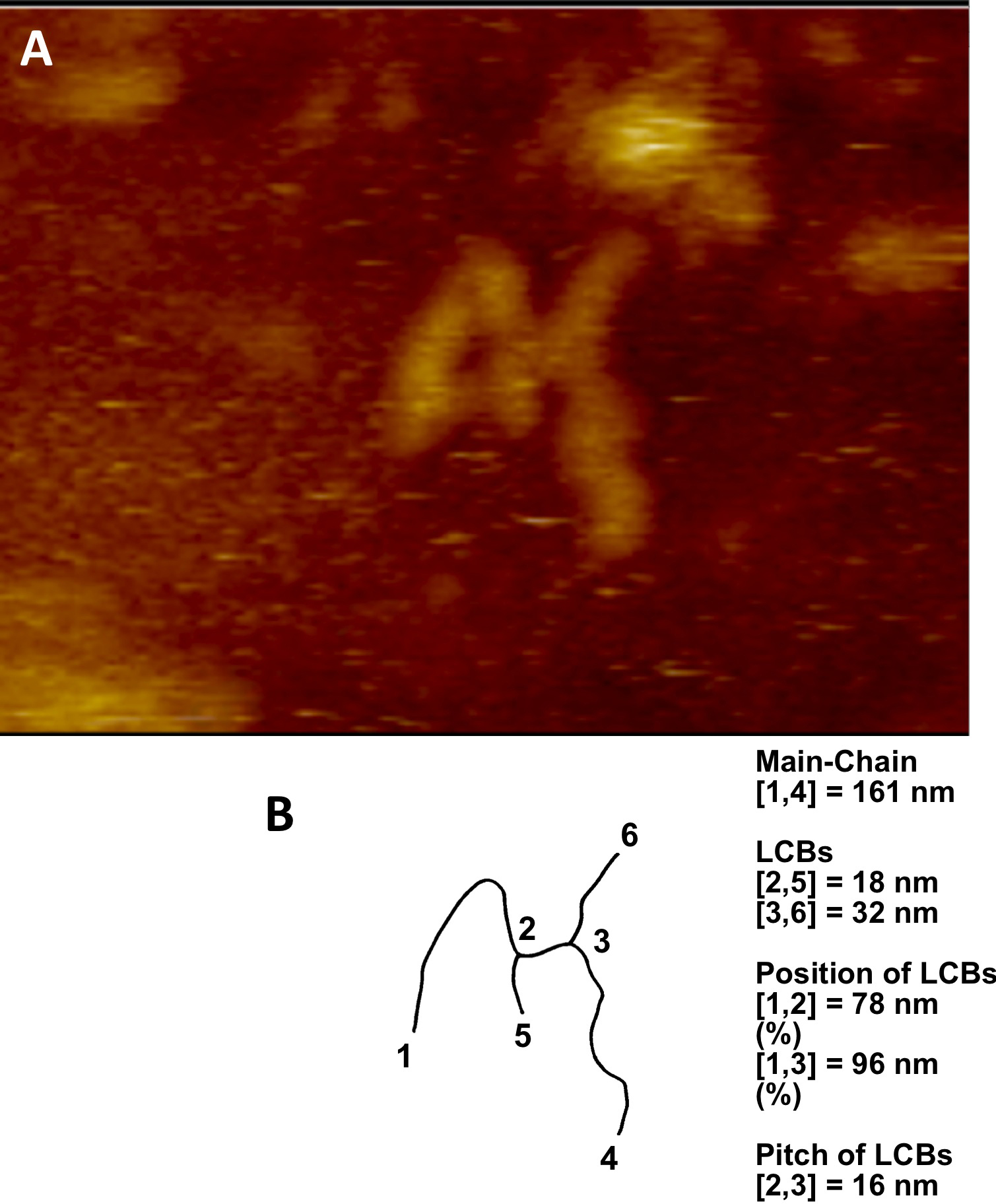
**

**Fig. S2.** Direct measurement of LCB in a tubular LDPE. **(A)** AFM image of a single molecule of LDPE on mica in DMTS at 25°C. X: 300 nm, Y: 225 nm, Z: 18 nm. **(B)** Length of each chain of LDPE.

The structure of single polymer chain of LDPE was directly observed in *n*-octylbenzene on mica substrate by AFM (Fig. S3A). The length measured by AFM (Fig. S3B) is calculated as (Main Chain)/(Main Chain + LCBs) = 228 nm/(228 nm + 28 nm + 48 nm + 40 nm) = 0.663.


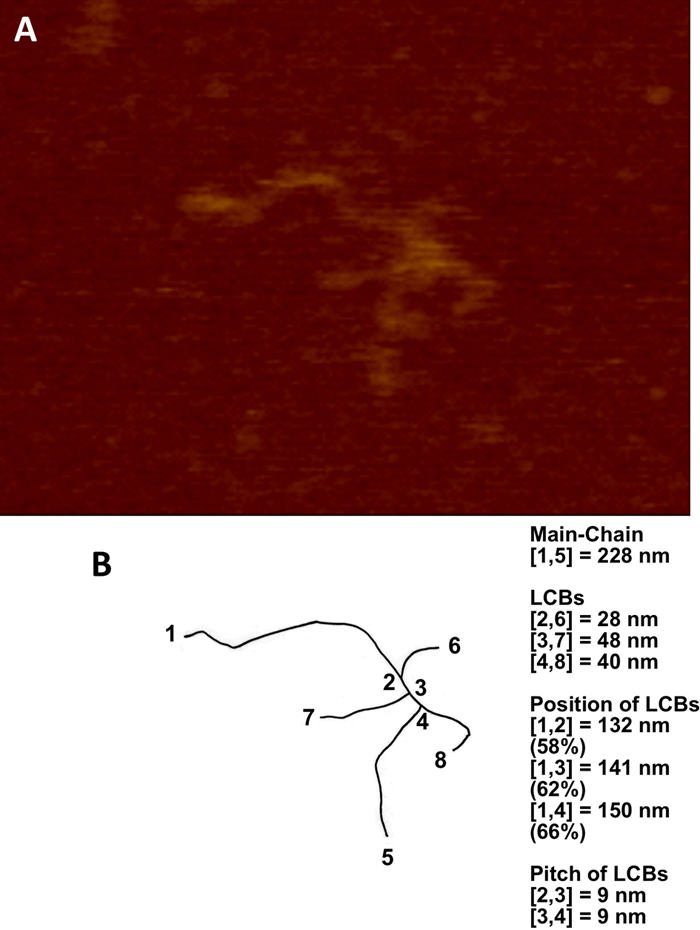


**Fig. S3.** Direct measurement of LCB in a tubular LDPE. **(A)** AFM image of a single molecule of LDPE on mica in *n*-octylbenzene at 25°C. X: 290 nm, Y: 218 nm, Z: 7.2 nm. **(B)** Length of each chain of LDPE.

**Supplementary References**

1. Shinohara, K. *Jpn. Pat.,* P5907484 (2016).
2. Shinohara, K. Pat., WO 2014104172 A1 (2014).
3. Ando, T., Kodera, N., Takai, E., Maruyama, D., Saito, K. & Toda, A. *Proc. Natl. Acad. Sci. USA* **98,** 12468 (2001).
4. Ando, T. Nanotechnology **23,** 062001 (2012).
